# Editorial: Advances in heart valve imaging

**DOI:** 10.3389/fcvm.2024.1450661

**Published:** 2024-07-11

**Authors:** Konstantinos Papadopoulos, Luigi P. Badano, Mani A. Vannan, Matteo Cameli, Anna Palmisano, Francesco Ancona, Antonio Esposito

**Affiliations:** ^1^ Echocardiography Department, European Interbalkan Medical Center, Thessaloniki, Greece; ^2^ Department of Medicine and Surgery, University of Milano Bicocca, Milan, Italy; ^3^ Department of Cardiology, Istituto Cardiologico Italiano, IRCCS, Milan, Italy; ^4^ Structural and Valvular Center of Excellence, Marcus Heart Valve Center, Piedmont Heart Institute, Atlanta, GA, United States; ^5^ Department of Medical Biotechnology, University of Siena, Siena, Italy; ^6^ School of Medicine, Vita Salute San Raffaele University, Milan, Italy; ^7^ Unit of Advanced Imaging for Personalized Medicine, IRCCS Ospedale San Raffaele, Milan, Italy; ^8^ Unit of Cardiovascular Imaging, IRCCS San Raffaele Scientific Institute, Milan, Italy

**Keywords:** heart valves, imaging, transcatheter interventions, artificial intelligence, structural heart diseases

**Editorial on the Research Topic** Advances in heart valve imaging

Heart valve diseases (HVD) pose significant challenges for imaging specialists, especially when combined with other cardiac pathologies. The advent of transcatheter interventions necessitates a multidisciplinary “heart team,” including a skilled multi-modality imaging department. This team assesses patient suitability for various treatments, guides operations, and evaluates final results. It is now recognized that beyond the anatomical characteristics of the valves, parameters such as ventricular and atrial dimensions and performance, pulmonary pressures, and other comorbidities must be evaluated during pre-operative screening for valvulopathies.

Recent advances in three-dimensional echocardiography, 2D speckle-tracking, and cardiac CT and MRI have enhanced our understanding of the pathophysiology of a severe valvulopathy. Additionally, artificial intelligence (AI) and machine learning are becoming significant in screening and managing patients with HVD. This special issue explores the advances in imaging for HVD and the potential role of AI in the near future (Table 1).

**Table 1:** Summary of all articles with authors and main findings included in this special issue.

Authors	Title	Summary
Aortic Stenosis – TAVI		
Xiao et al.	Determinants of device success after transcatheter aortic valve replacement in patients with type-0 bicuspid aortic stenosis	Bulky calcifications of AOV commissures play a negative role in the device success after TAVI in type-0 BAV
Parasca et al.	Right ventricle to pulmonary artery coupling after transcatheter aortic valve implantation-Determinant factors and prognostic impact	RV-PA coupling is improving after TAVI except for patients with persistent pulmonary hypertension. Baseline measurements of RV-PA coupling can provide information about patients’ outcomes
De la Torre Hernandez et al.	First description and validation of a new method for estimating aortic stenosis burden and predicting the functional response to TAVI	LVOT flow velocity and aortic pressure loops can predict the functional improvement after TAVI through the parameter “[P (Vmax) − P (Vo)]/Vmax and lead to better quality-of life post-procedure
Pestiaux et al.	3D histopathology of stenotic aortic valve cusps using ex vivo microfocus computed tomography	High-resolution microfocus-CT can define the AOV calcification burden and cusps thickness. Aortic valves have thinner cusps with less calcium in low-gradient stenosis rather in high-gradient.
Namasivayam et al.	Machine learning prediction of progressive subclinical myocardial dysfunction in moderate aortic stenosis	AI identified peak AOV gradient, dimensionless index, baseline GLS and energy loss as predictors of subclinical myocardial dysfunction progression in moderate AS.
Mitral valve pathology		
Apostolou et al.	Case report: Aborted sudden cardiac death as a first presentation of severe mitral annulus disjunction-a case series and review of the literature	Cardiac MRI is important in detecting fibrosis as a cause of malignant arrhythmias and SCD in MAD syndrome
Papadopoulos et al.	The added value of three-dimensional transthoracic echocardiography in mitral annular disjunction: a case report	Three-dimensional echocardiography can provide most echocardiographic features of MAD syndrome and detect the presence of fibrosis through 4D strain analysis.
Zheng et al.	Understanding post-surgical decline in left ventricular function in primary regurgitation using regression and machine learning models	AI can detect the decline of EF after MV repair in patient with primary MR. EF, Sphericity index, LV end-systolic diameter and circumferential strain rate detected a post-operative EF < 50% in the random forest model.
Mihos	Left ventricular remodeling, mechanics, and the COAPT trial	COAPT criteria should be followed in patients with SMR and HFrEF but baseline GLS can further predict the LV remodeling after TEER and should be evaluated
HVD and coronary artery disease		
Molenaar et al.	The impact of valvular heart disease in patients with chronic coronary syndrome	A thorough echocardiographic examination should be conducted in all patients with CAD since HVD and especially moderate TR indicate myocardial damage and a poorer prognosis in these patients
Multimodality imaging and complex cardiac surgery		
Huenges et al.	Case report: The woman with the big heart-an imaging-guided attempt of surgical reduction	Multimodality imaging guided a complete pre-operative planning of a case that demanded atrial reduction along with MV replacement and CABG with a successful result

Transcatheter Aortic Valve Implantation (TAVI) is the most common transcatheter intervention with expanding indications, garnering attention from the cardiology community. Xiao et al. studied a small cohort of patients with type-0 bicuspid aortic valve and identified predictors of success after TAVI. They found that the ellipticity index of the aortic root and bulky calcifications of aortic commissures differed between success and failure subgroups. Bulky calcifications were determined by visual assessment using multidetector CT transverse planes and maximum intensity projections. Multivariate analysis revealed though that only bulky calcifications had negative correlation with device success post-TAVI. This study highlights the importance of calcification degree and distribution in managing patients with bicuspid aortic valve, suggesting that a larger patient series could further improve TAVI outcomes for this group.

Right ventricular (RV) dysfunction and pulmonary hypertension are crucial in determining TAVI outcomes. Parasca et al. showed that RV-pulmonary artery (PA) coupling improves after TAVI, except in patients with persistent pulmonary hypertension. They emphasized the importance of evaluating RV-PA coupling during TAVI screening. A baseline RV-free wall longitudinal strain (FWLS)/pulmonary artery systolic pressure (PASP) cutoff of 0.63 was able to differentiate between normal and impaired RV-PA coupling, providing valuable prognostic information and guiding treatment decisions to improve patient outcomes.

Predicting the response after TAVI is vital since 20% of patients continue to experience poor quality of life post-procedure. De la Torre Hernandez et al. validated a new method to assess aortic stenosis (AS) burden and functional outcome after TAVI. This method integrates left ventricular outflow tract flow velocity and aortic pressure, focusing on parameters like “[*P* (Vmax) − *P* (Vo)]/Vmax,” an independent predictor of functional improvement post-TAVI. This approach offers a more comprehensive hemodynamic assessment, potentially leading to better patient outcomes and quality of life post-procedure.

Understanding aortic stenosis mechanisms is crucial for treatment and patient outcomes. Pestiaux et al. analyzed the microstructure of calcified aortic valve cusps using high-resolution microfocus CT, demonstrating the calcification burden and cusp thickness. They found that aortic valves have thinner cusps with significantly less calcium in low-gradient vs. high-gradient patients. While not routine, this examination can enhance *in vivo* imaging protocols and clinical data interpretation.

AI plays a significant role in diagnosing structural heart diseases. Namasivayam et al. applied artificial neural networks to echocardiographic data from patients with moderate AS, identifying predictors of subclinical myocardial dysfunction progression. Key factors include peak gradient, dimensionless index (DI), baseline left ventricular global longitudinal strain (GLS), and energy loss. These parameters should be closely monitored in patients with moderate AS.

Mitral valve pathology, including new entities like mitral annular disjunction (MAD), is also under extensive research. Apostolou et al. described cases of aborted sudden cardiac death in patients with mitral valve prolapse and MAD, investigated using cardiac MRI, which revealed fibrosis associated with malignant arrhythmias. This highlights the significance of multimodality imaging and interdisciplinary collaboration for managing such patients. Papadopoulos et al. suggested that three-dimensional transthoracic echocardiography and 4D strain analysis might suffice to demonstrate MAD features and fibrosis presence, but this needs validation through larger studies.

AI has been tested in mitral valve diseases as well. Zheng et al. used regression and machine learning models to predict postoperative ejection fraction (EF) decline in primary mitral regurgitation (PMR) patients. The random forest model accurately detected patients with postoperative EF < 50%, including predictors like LVEF, LV sphericity index, LV end-systolic diameter (LVESD), and LV mid-systolic circumferential strain rate. Although further research is needed, this study suggests a more accurate preoperative assessment for these patients.

LV mechanics are crucial for outcomes after transcatheter edge-to-edge repair (TEER) in secondary mitral regurgitation (SMR) patients. Mihos emphasized the importance of GLS in predicting left ventricular remodeling, crucial for patient prognosis. Following the echocardiographic criteria from the COAPT trial, which demonstrated TEER benefits in heart failure with reduced ejection fraction (HFrEF) and SMR, is recommended. However, deformation metrics might provide additional data on TEER responders. Heart teams managing these patients should also consider guideline-directed medical therapy and cardiac resynchronization therapy (CRT) when indicated.

In a study of about 2,000 patients, Molenaar et al. found that HVD affect prognosis in coronary artery disease (CAD) patients. LV dysfunction and moderate or severe HVD were the main indicators of higher mortality. Moderate tricuspid regurgitation (TR) was the strongest mortality predictor in multivariable regression analysis. HVD often indicates a higher atherosclerotic burden and more significant CAD, leading to more common myocardial damage and poorer prognosis. A thorough echocardiographic examination should be conducted when screening CAD patients.

Cardiac surgery has also progressed, allowing for the efficient management of complex cases. Huenges et al. described treating a patient with a gigantic left atrium requiring mitral valve replacement and coronary artery bypass grafting (CABG). Thorough multi-modality imaging with echocardiography and cardiac CT facilitated a successful operation, including atrial reduction techniques and MV replacement.

In summary, advances in imaging and AI are transforming the management of HVD and improve patients’ outcomes through more accurate preoperative assessments and innovative treatment approaches.

